# Estrogen Status and Temporomandibular Disorders: A Systematic Review

**DOI:** 10.3390/ijerph23060717

**Published:** 2026-05-28

**Authors:** Alexandru Mazareanu, Claudia Grigorov, Alin Pandea, Maria Iacob, Dragos George Balaiasa, Tzvika Greenbaum, Petr Konecny

**Affiliations:** 1 Department of Physical Therapy, University of Medical Sciences Arizona (UMSAZ), Honolulu, HI 96813-3481, USA; a_mazareanu@yahoo.com; 2 Department of Endocrinology, Medlife Hospital, 900001 Constanța, Romania; 3 Regina Maria Hospital and Nord Hospital, 011171 Bucharest, Romania; alinpandea@gmail.com; 4 Private Dental Practice, 700506 Iași, Romania; 5 Faculty of Physical Education and Sports, “Alexandru Ioan Cuza” University, 700506 Iași, Romania; 6 Department of Physical Therapy, Faculty of Health Sciences, Recanati School for Community Health Professions, Ben-Gurion University of the Negev, Be’er-Sheva 8410501, Israel; 7 Department of Physiotherapy, Faculty of Health Sciences, Palacký University Olomouc, 779 00 Olomouc, Czech Republic; petr.konecny@upol.cz

**Keywords:** temporomandibular disorders, estrogen, hormonal contraceptives, menopause, systematic review

## Abstract

**Highlights:**

**Public health relevance—How does this work relate to a public health issue?**

- Temporomandibular disorders disproportionately affect women and represent a significant cause of chronic pain and functional impairment, making sex-specific risk factors a relevant public health issue.
- This review addresses the role of estrogen-related factors, including hormonal contraceptive use and menopausal status, in the risk and clinical presentation of TMD.

**Public health significance—Why is this work of significance to public health?**

- The findings highlight the importance of hormonal status as a potential determinant of chronic musculoskeletal and orofacial pain in women.
- Understanding these associations may support earlier identification of women at increased risk of TMD during reproductive and menopausal transitions.

**Public health implications—What are the key implications or messages for practitioners, policy makers and/or researchers in public health?**

- Clinicians and public health practitioners should consider hormonal transitions and reproductive health status when assessing and managing women with TMD symptoms.
- The review underscores the need for prospective population-based studies and standardized diagnostic protocols to guide evidence-based women’s health, pain management, and headache-related rehabilitation strategies.

**Abstract:**

Background: Temporomandibular disorders (TMDs) exhibit a marked female predominance, suggesting a potential role for estrogen in their pathophysiology. However, evidence linking estrogen status to TMD remains inconsistent. Objective: To systematically review the association between estrogen-related factors and TMD prevalence and clinical presentation in women. Methods: PubMed, Embase, Scopus, Web of Science, and Google Scholar were searched through September 2025. Observational studies evaluating hormonal contraceptive use, menopausal status, menstrual cycle variation, pregnancy, or estrogen receptor polymorphisms in women with TMD were included. Two reviewers independently performed study selection, data extraction, and risk-of-bias assessment using the Newcastle-Ottawa Scale. Due to substantial heterogeneity, a narrative synthesis was conducted. Results: Seven studies met the inclusion criteria, including six clinical studies involving 2735 participants and one mechanistic supportive study. Moderate-certainty evidence suggested associations between hormonal contraceptive use, menopausal/climacteric status, and increased TMD risk or symptom severity. Additional low-certainty evidence supported associations involving menstrual cycle variation, pregnancy, and estrogen receptor polymorphisms. Conclusions: Current evidence suggests that hormonal factors may influence TMD risk and symptom presentation in women. However, heterogeneity in definitions of hormonal exposure and diagnostic criteria limits definitive conclusions. Further prospective studies using standardized diagnostic protocols and real-time biochemically validated hormonal assessments correlated with clinical symptoms are needed.

## 1. Introduction

Temporomandibular disorders (TMDs) comprise a heterogeneous group of musculoskeletal conditions affecting the temporomandibular joint (TMJ), the masticatory muscles, and associated craniofacial structures, and are among the leading causes of chronic orofacial pain worldwide [1,2]. The estimated global prevalence ranges between 5% and 12%, with substantial functional, psychosocial, and economic consequences [2]. A consistent and striking epidemiological feature of TMD is its marked female predominance, with women affected approximately two to four times more frequently than men, particularly during reproductive and perimenopausal years [2,3]. This sex disparity has long suggested a potential role for hormonal factors—most notably estrogen—in TMD pathophysiology [4].

Estrogen is a key regulator of pain processing, connective tissue metabolism, and inflammatory responses. Both estrogen receptor alpha (ERα) and beta (ERβ) have been identified in TMJ synovium, fibrocartilage, subchondral bone, and masticatory muscles, indicating that estrogen signaling may directly influence joint and muscular homeostasis [5,6]. Experimental and translational studies suggest that estrogen modulates chondrocyte viability, collagen turnover, synovial lubrication, and the expression of pro-inflammatory cytokines such as interleukin-6 (IL-6) and tumor necrosis factor-alpha (TNF-α) [6,7,8]. Fluctuations or declines in estrogen levels—such as those occurring during the menstrual cycle, menopause, or hypoestrogenic states—may therefore increase susceptibility to joint degeneration, synovial inflammation, and enhanced nociceptive transmission, all of which are central features of TMD.

Clinical observations support these biological mechanisms. Several studies have reported symptom exacerbation during periods of hormonal fluctuation, including menstruation and the menopausal transition, as well as altered TMD prevalence in women receiving hormone replacement therapy (HRT) or oral contraceptives (OCs) [9,10,11,12,13]. However, findings across studies remain inconsistent. While some investigations suggest that estrogen deficiency or absence of HRT increases TMD risk and pain severity, others report neutral or heterogeneous associations, particularly with respect to OC use [10]. Differences in study design, diagnostic criteria, methods of hormonal assessment, and population characteristics have contributed to ongoing uncertainty regarding the magnitude and clinical relevance of estrogen’s role in TMD [3].

Beyond joint-specific mechanisms, emerging neuromuscular and biomechanical models propose that estrogen may influence pain sensitivity and motor control within the broader cervico-cranio-mandibular system. Sex-related differences in masticatory and cervical muscle performance, coordination, and pain thresholds have been described in individuals with TMD, suggesting that hormonal modulation may extend beyond local joint tissues to central and peripheral neuromuscular regulation [14,15,16]. These findings align with contemporary views of TMD as a complex, multisystem disorder characterized by interactions among endocrine, musculoskeletal, and neurophysiological factors.

Despite a growing body of observational and experimental research, the overall evidence linking estrogen status, hormonal therapies, and TMD remains fragmented. Previous systematic reviews have been limited by narrow inclusion criteria, small sample sizes, inconsistent outcome measures, or the absence of quantitative synthesis, which precludes robust estimation of effect sizes and limits clinical interpretation [3,17]. To date, no comprehensive systematic review has integrated both clinical and hormonal data across diverse female populations with rigorous quality assessment.

Temporomandibular disorders frequently coexist with primary and secondary headache disorders, including migraine and cervicogenic headache [18,19], through shared trigeminal and cervico-cranial nociceptive pathways. Given the marked female predominance and hormonal sensitivity observed in both TMD and headache disorders, understanding the role of estrogen in TMD may also provide broader insights into headache-related pain modulation and rehabilitation strategies in women.

Therefore, the aim of this systematic review was to provide a comprehensive synthesis of the available evidence on the relationship between estrogen status—including circulating estrogen levels, menopausal status, hormonal contraceptive use, menstrual cycle variation, pregnancy, and estrogen receptor gene polymorphisms—and the prevalence and clinical features of temporomandibular disorders in women. We hypothesized that lower estrogen levels and the absence of hormonal supplementation would be associated with increased TMD risk and symptom severity.

## 2. Methods

### 2.1. Protocol and Registration

This systematic review was conducted in accordance with the Preferred Reporting Items for Systematic Reviews and Meta-Analyses (PRISMA) 2020 guidelines [20].

The protocol for this systematic review was prospectively registered in the International Prospective Register of Systematic Reviews (PROSPERO; CRD420251154149). Available online: https://www.crd.york.ac.uk/PROSPERO/view/CRD420251154149 (accessed on 16 May 2026). The PRISMA 2020 checklist is provided in Supplementary Table S1.

### 2.2. Eligibility Criteria

Studies were included if they met the following criteria:

Population: Adult women (≥18 years) diagnosed with TMD using validated diagnostic criteria, including the Research Diagnostic Criteria for TMD (RDC/TMD), Diagnostic Criteria for TMD (DC/TMD), or equivalent clinical examination protocols.

Exposure: Any estrogen-related variable, including: Hormonal contraceptive use (oral contraceptives, hormonal intrauterine devices, transdermal patches)—Menopausal or climacteric status (premenopausal, perimenopausal, postmenopausal)—Menstrual cycle phase—Pregnancy status—Hormone replacement therapy (HRT)—Circulating estradiol levels—Estrogen receptor gene polymorphisms.

Comparator: Women without the specified hormonal exposure or women in different hormonal states (e.g., premenopausal vs. postmenopausal).

Outcome: TMD prevalence, incidence, symptom severity, pain intensity, functional limitation, or TMJ structural changes assessed through clinical examination, validated questionnaires, or imaging.

Study Design: Observational studies (cohort, case–control, cross-sectional, and prospective observational diary studies). In addition, mechanistic human ex vivo studies were considered for supportive biological evidence when directly relevant to estrogen-related inflammatory pathways in TMD.

Exclusion Criteria: Exclusion criteria included studies not reporting primary data (e.g., reviews, editorials, or commentaries), studies with exclusively male participants, studies using non-validated TMD diagnostic criteria, studies not reporting estrogen-related exposures, animal studies, and purely non-human in vitro investigations. Human mechanistic ex vivo studies were considered only when they provided directly relevant supportive biological evidence regarding estrogen-related inflammatory or pain pathways in TMD; however, such studies were not included in the main clinical synthesis, risk-of-bias assessment, or GRADE certainty evaluation. Studies published in languages other than English were excluded to ensure methodological consistency and accurate interpretation of study findings during data extraction and quality assessment.

### 2.3. Information Sources and Search Strategy

A comprehensive literature search was conducted in three electronic databases from inception through September 2025: PubMed/MEDLINE, Scopus, and Web of Science Core Collection. In addition, Google Scholar was searched as a supplementary source to identify potentially eligible studies not indexed in the primary databases and to minimize the risk of publication bias.

The database searches were performed between September and October 2025 and included studies published through September 2025. The search strategy combined Medical Subject Headings (MeSH) terms and free-text keywords related to temporomandibular disorders, estrogen, hormonal status, and related hormonal exposures, including hormonal contraceptive use, menopausal status, menstrual cycle variation, pregnancy, and estrogen receptor polymorphisms. Search strategies were adapted as appropriate for the syntax and indexing systems of each database. 

Reference lists of included studies and relevant review articles were also hand-searched to identify additional eligible studies. Full search strategies for all databases are provided in Supplementary Table S2.

### 2.4. Study Selection

**Figure 1:**
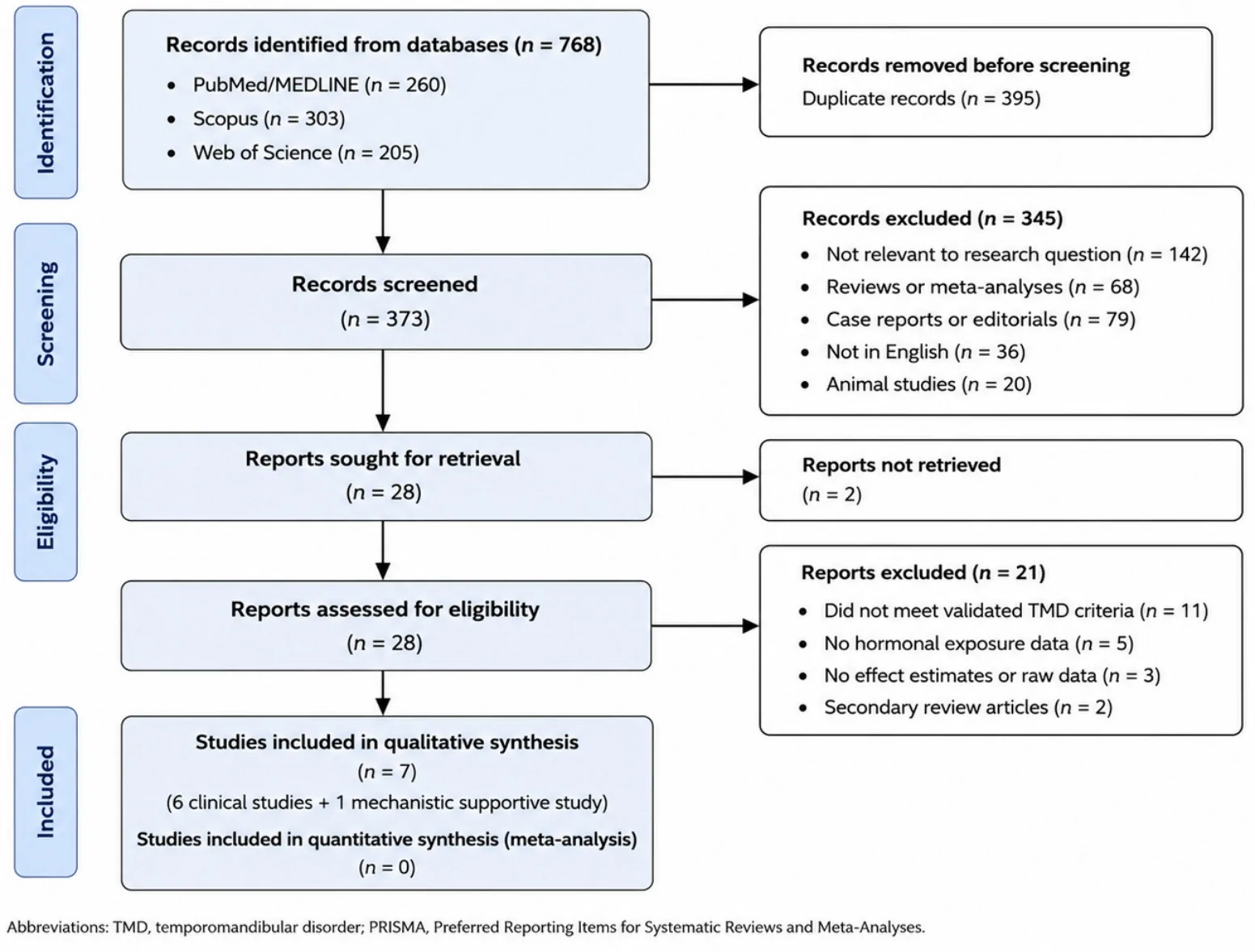
PRISMA 2020 flow diagram of the study selection process.

Two independent reviewers (AM and CG) screened titles and abstracts for eligibility. Full-text articles of potentially eligible studies were retrieved and assessed against the inclusion criteria. Disagreements were resolved through discussion or consultation with a third reviewer (TG). The study selection process is summarized in a PRISMA flow diagram (Figure 1).

### 2.5. Data Extraction

Data were extracted independently by two reviewers (AM and MI) using a standardized form. The following information was recorded:—Study characteristics: first author, year, country, study design, sample size—Participant characteristics: age, TMD diagnostic criteria, hormonal exposure definition—Outcomes: TMD prevalence, incidence, symptom severity, effect estimates (odds ratios, risk ratios, hazard ratios with 95% confidence intervals)—Adjustments: covariates included in multivariable models.

Disagreements were resolved by consensus.

### 2.6. Risk of Bias Assessment

The methodological quality of included studies was assessed using the Newcastle-Ottawa Scale (NOS) for observational studies [21]. The NOS evaluates three domains: selection of study groups (0–4 stars), comparability of groups (0–2 stars), and ascertainment of exposure/outcome (0–3 stars). Scores of ≥7 stars were considered high quality, 5–6 stars moderate quality, and <5 stars low quality. Two reviewers (AP and DB) independently assessed risk of bias, with discrepancies resolved through discussion.

### 2.7. Data Synthesis

Due to substantial heterogeneity in exposure definitions (hormonal contraceptive use, menopausal status, menstrual cycle phase, pregnancy, genetic polymorphisms), outcome measures (TMD prevalence, first-onset TMD, palpation pain, crepitus, degenerative joint disease), and study designs (prospective cohort, cross-sectional, case–control), a narrative synthesis was performed. Studies were grouped by hormonal exposure category, and findings were summarized descriptively.

Considerable heterogeneity was observed across studies in hormonal exposure definitions (e.g., oral contraceptive formulations, menopausal criteria), TMD diagnostic approaches (RDC/TMD, DC/TMD, modified DC/TMD, or clinical assessment), and outcome measures, which limited comparability across studies and further supported the decision to perform a narrative rather than quantitative synthesis.

Meta-analysis was not conducted because 1. Only one study per hormonal exposure category reported extractable effect estimates with 95% confidence intervals. 2. Exposure definitions were not sufficiently homogeneous to permit pooling (e.g., hormonal contraceptive use vs. climacteric status). 3. Outcome definitions varied substantially across studies (first-onset TMD vs. palpation pain vs. degenerative joint disease). 4. The minimum requirement of at least two studies per outcome for meta-analysis was not met.

In addition, the available evidence base was characterized by substantial variability in study sample sizes, ranging from small exploratory studies to large-scale cohort investigations, with a considerable proportion of participants derived from a limited number of large studies. This imbalance should be considered when interpreting the overall strength and generalizability of the findings.

The certainty of evidence was assessed using the Grading of Recommendations Assessment, Development and Evaluation (GRADE) approach [22], considering risk of bias, inconsistency, indirectness, imprecision, and publication bias.

## 3. Results

### 3.1. Study Selection

The reconstructed literature search identified 768 records across PubMed/MEDLINE, Scopus, and Web of Science databases. After removing 395 duplicate records, 373 unique records remained for title and abstract screening. Following screening, 28 full-text articles were assessed for eligibility, and 7 studies [23,24,25,26,27,28,29] met the inclusion criteria and were included in the qualitative synthesis. Due to substantial heterogeneity in exposures, study designs, and outcomes, no quantitative meta-analysis was performed.

### 3.2. Study Characteristics

Overall, seven studies were included, comprising six clinical studies involving 2735 participants and one mechanistic supportive study involving 18 participants. The included studies were published between 2003 and 2024. Study designs comprised one prospective cohort study, two case–control studies, two cross-sectional studies, one prospective diary study, and one mechanistic human ex vivo study included as supportive biological evidence. The studies were conducted in the United States (*n* = 3), Finland (*n* = 1), Italy (*n* = 1), and Indonesia (*n* = 1), with the mechanistic supportive study conducted in the United States as well. TMD was diagnosed using RDC/TMD (*n* = 3), DC/TMD (*n* = 1), modified DC/TMD (*n* = 1), or clinical TMD assessment (*n* = 1). Hormonal exposures examined included hormonal contraceptive use (*n* = 2), menopausal/climacteric status (*n* = 2), menstrual cycle phase (*n* = 1), pregnancy (*n* = 1), serum estradiol levels (*n* = 1), and estrogen receptor gene polymorphisms (*n* = 1), with one mechanistic study evaluating estrogen-induced inflammatory signaling pathways. Detailed characteristics of clinical studies are presented in Table 1.

### 3.3. Risk of Bias Assessment

Risk of bias assessment using the Newcastle-Ottawa Scale revealed that four studies were of high methodological quality (NOS ≥ 7) [23,24,25,27] and two studies were of moderate quality (NOS 5–6) [26,28]. No study was rated as low quality. Common methodological limitations included the lack of biochemical validation of hormonal status in some studies, reliance on self-reported hormonal exposures, small sample sizes in cross-sectional and case–control designs, and limited adjustment for potential confounders such as body mass index, smoking status, and psychosocial factors. The mechanistic supportive study was not included in the risk-of-bias assessment, as the Newcastle-Ottawa Scale is not applicable to ex vivo mechanistic designs. Results of the risk-of-bias assessment are summarized in Table 2.

### 3.4. Evidence Synthesis by Hormonal Exposure Category

#### 3.4.1. Hormonal Contraceptive Use and TMD

Two studies examined the association between hormonal contraceptive use and TMD [23,25]. Gaynor et al. (2021) conducted a high-quality prospective cohort study within the Orofacial Pain: Prospective Evaluation and Risk Assessment (OPPERA) study, involving 1475 women aged 18–44 years [23]. Hormonal contraceptive use was associated with increased odds of self-reported first-onset TMD pain symptoms (OR 1.37, 95% CI 1.13–1.66) and concurrent self-reported TMD symptoms (OR 1.20, 95% CI 1.06–1.35). However, hormonal contraceptive use was not significantly associated with examiner-classified incident TMD (HR 1.36, 95% CI 0.82–2.24) after adjustment for confounders. LeResche et al. (2003) reported cyclic variation in TMD symptoms across the menstrual cycle in a prospective diary study of 70 TMD-affected women, with symptoms peaking during menstruation and ovulation; however, oral contraceptive use did not significantly modify this pattern [25].

GRADE Assessment: The certainty of the evidence for hormonal contraceptive use and TMD was rated as moderate, downgraded due to indirectness (a single study) and imprecision (wide confidence intervals in one study).

#### 3.4.2. Menopausal/Climacteric Status and TMD

Two studies investigated the relationship between menopausal or climacteric status and TMD [24,28]. Mursu et al. (2022) conducted a high-quality cross-sectional study within the Northern Finland Birth Cohort 1966, involving 727 women aged 46 years [24]. Climacteric status, defined as amenorrhea >4 months plus follicle-stimulating hormone (FSH) >25 IU/L, was significantly associated with increased risk of TMJ palpation pain (OR 2.64, 95% CI 1.12–6.21), crepitus (OR 2.92, 95% CI 1.13–7.56), and degenerative joint disease (OR 2.27, 95% CI 1.05–4.91) after adjustment for body mass index, smoking, and parity.

Rosanto et al. (2020) found higher mean serum estradiol levels in postmenopausal women with anterior disc displacement compared to those with normal TMJ (50.54 ± 33.93 vs. 32.14 ± 22.51 pg/mL), but the difference was not statistically significant (*p* > 0.05) [28].

GRADE Assessment: The certainty of evidence for menopausal/climacteric status and TMD was rated as moderate, downgraded due to inconsistency (heterogeneous outcome definitions) and imprecision (wide confidence intervals).

#### 3.4.3. Menstrual Cycle Variation in TMD Symptoms

One high-quality prospective diary study by LeResche et al. (2003) documented cyclic variation in TMD symptom severity across three menstrual cycles in 70 TMD-affected women [25]. Pain intensity was highest during menstruation, with a secondary peak during the estimated ovulation phase. This pattern was observed regardless of oral contraceptive use, suggesting that endogenous hormonal fluctuations influence symptom expression.

GRADE Assessment: The certainty of the evidence for menstrual cycle variation and TMD was rated as low, downgraded due to indirectness (a single study) and imprecision (small sample size).

#### 3.4.4. Pregnancy and TMD

One moderate-quality cross-sectional study by Minervini et al. (2024) compared chronic pain grades between 32 pregnant and 35 non-pregnant women aged 18–40 years using DC/TMD Axis II measures [26]. Pregnant women exhibited lower chronic pain grades (β = −0.67, *p* = 0.032); however, this association did not remain significant after correction for multiple comparisons. The study was limited by a small sample size and a cross-sectional design.

GRADE Assessment: The certainty of evidence for pregnancy and TMD was rated as very low, downgraded due to serious imprecision (small sample size, loss of significance after correction) and indirectness (single study).

#### 3.4.5. Estrogen Receptor Gene Polymorphisms and TMD

One high-quality case–control genetic association study examined the relationship between estrogen receptor-α (ERα) gene polymorphisms and TMD susceptibility [27]. Ribeiro-Dasilva et al. (2009) included 300 women (100 with painful TMJD, 100 with painless TMJD, and 100 controls) and reported that the XbaI and PvuII polymorphisms in the ERα gene were significantly associated with both painful TMJD (OR 3.20, 95% CI 1.63–6.28) and painless TMJD (OR 2.51, 95% CI 1.27–4.97) compared with controls, suggesting a potential genetic susceptibility mediated by estrogen receptor signaling [27].

In addition, Ribeiro-Dasilva et al. (2017) demonstrated that ex vivo estrogen stimulation of monocytes obtained from women with and without TMD included increased interleukin-6 (IL-6) production, and that these inflammatory responses were associated with pain measures among women with TMD [29]. Although this mechanistic study was not included in the main clinical synthesis, it provides important biological plausibility supporting the role of estrogen-related inflammatory pathways in TMD pathophysiology.

GRADE Assessment: The certainty of evidence for estrogen receptor gene polymorphisms and related mechanistic evidence was rated as low, downgraded due to indirectness, as genetic susceptibility and ex vivo inflammatory responses do not directly reflect clinical hormonal exposure status.

### 3.5. Summary of Evidence by Exposure Category

A summary of the evidence by hormonal exposure category, including the number of studies, quality of evidence, and GRADE certainty ratings, is presented in Table 3.

## 4. Discussion

### 4.1. Principal Findings

This systematic review synthesized evidence from seven studies, comprising six clinical studies involving 2735 participants and one mechanistic supportive study involving 18 participants, to evaluate the association between estrogen status and temporomandibular disorders in women. The principal findings indicate that hormonal factors, particularly hormonal contraceptive use and menopausal/climacteric status, are associated with increased TMD risk and altered symptom presentation. High-quality evidence from prospective cohort and cross-sectional studies demonstrated that hormonal contraceptive use was associated with increased odds of self-reported first-onset TMD pain symptoms (OR 1.37, 95% CI 1.13–1.66) and concurrent self-reported TMD symptoms (OR 1.20, 95% CI 1.06–1.35), whereas no significant association was observed for examiner-classified incident TMD (HR 1.36, 95% CI 0.82–2.24). In contrast, climacteric status was associated with increased risk of TMJ palpation pain (OR 2.64, 95% CI 1.12–6.21), crepitus (OR 2.92, 95% CI 1.13–7.56), and degenerative joint disease (OR 2.27, 95% CI 1.05–4.91). Additional evidence supported variation in menstrual cycle-related symptoms and genetic susceptibility mediated by estrogen receptor polymorphisms. However, the limited number of high-quality studies, heterogeneity in exposure definitions, and variability in diagnostic criteria constrain definitive conclusions regarding causality and clinical implications.

### 4.2. Interpretation of Findings by Exposure Category

#### 4.2.1. Hormonal Contraceptive Use

The association between hormonal contraceptive use and increased TMD risk observed in the OPPERA cohort [23] contrasts with the hypothesis that exogenous estrogen supplementation might be protective. This paradoxical finding may be explained by several mechanisms. First, synthetic progestins in combined oral contraceptives may exert pro-inflammatory effects independent of estrogen, potentially increasing joint inflammation and pain sensitivity [30]. Second, hormonal contraceptives may alter endogenous estrogen receptor expression or signaling pathways, thereby dysregulating inflammatory responses in TMJ tissues [31]. Third, individual variability in estrogen metabolism and receptor polymorphisms may modulate the net effect of exogenous hormones on TMD susceptibility [28]. The cyclic variation in symptoms observed by LeResche et al. [25], which persisted regardless of oral contraceptive use, suggests that endogenous hormonal fluctuations play a dominant role in symptom modulation and that exogenous hormones may not fully override these patterns.

#### 4.2.2. Menopausal/Climacteric Status

The strong association between climacteric status and TMD observed by Mursu et al. [24] aligns with the biological hypothesis that estrogen deficiency increases susceptibility to joint degeneration and pain. Postmenopausal declines in estrogen reduce chondrocyte viability, impair collagen synthesis, and increase expression of matrix metalloproteinases (MMPs), all of which contribute to cartilage degradation and TMJ osteoarthritis [32]. Additionally, estrogen deficiency is associated with increased systemic inflammation, as evidenced by elevated circulating levels of IL-6 and TNF-α, which may amplify local inflammatory responses in TMJ tissues [33]. The biochemical validation of climacteric status using FSH levels in the Mursu study [24] strengthens confidence in these findings, as self-reported menopausal status is prone to misclassification.

The lack of significant association between serum estradiol levels and anterior disc displacement in the Rosanto study [28] may reflect the small sample size, cross-sectional design, and potential confounding by other hormonal and metabolic factors. Disc displacement is a structural outcome that may develop over years and may not be directly correlated with circulating estrogen levels at a single time point.

#### 4.2.3. Menstrual Cycle Variation

The cyclic variation in TMD symptoms documented by LeResche et al. [25] provides compelling evidence that endogenous hormonal fluctuations influence symptom expression. The peak in pain during menstruation, when estrogen and progesterone levels are lowest, is consistent with the hypothesis that estrogen withdrawal increases pain sensitivity and inflammatory responses. The secondary peak during ovulation, when estrogen levels are highest, suggests a more complex, non-linear relationship between estrogen levels and pain, possibly mediated by rapid hormonal shifts or interactions with other neuromodulators such as serotonin and endogenous opioids [34]. These findings underscore the importance of considering hormonal dynamics rather than static hormone levels when evaluating the role of estrogen in TMD.

#### 4.2.4. Pregnancy

The finding of lower chronic pain grades among pregnant women [26] is intriguing but must be interpreted with caution, as it lost statistical significance after correction for multiple comparisons. Pregnancy is characterized by sustained elevations in estrogen, progesterone, and relaxin, which may exert analgesic and anti-inflammatory effects [35]. However, pregnancy also involves substantial musculoskeletal adaptations, changes in pain perception, and psychosocial factors that may confound the relationship between hormonal status and TMD symptoms. Larger, longitudinal studies with validated TMD diagnostic criteria are needed to clarify this association.

#### 4.2.5. Estrogen Receptor Gene Polymorphisms

The association between ERα gene polymorphisms and TMD susceptibility [27] provides genetic evidence supporting a mechanistic role for estrogen signaling in TMD pathophysiology. The XbaI and PvuII polymorphisms may alter ERα expression or function, leading to differential responses to endogenous and exogenous estrogen [36]. The mechanistic findings reported by Ribeiro-Dasilva et al. [29] further support the hypothesis that estrogen modulates inflammatory responses in TMD-affected individuals through pathways involving IL-6 production. These findings suggest that genetic variability in estrogen receptor signaling may contribute to the observed heterogeneity in TMD susceptibility and symptom severity among women and may partially explain inconsistent findings across studies.

Although excluded from the main analysis due to its mechanistic (in vitro/ex vivo) design, Ribeiro-Dasilva et al. [29] provide important biological plausibility for the role of estrogen in TMD. The study demonstrated that estrogen stimulation of monocytes increased IL-6 production, which correlated with clinical pain intensity. These findings support the hypothesis that estrogen may modulate inflammatory pathways relevant to TMD pathophysiology.

### 4.3. Comparison with Previous Reviews

Previous systematic reviews on hormonal factors and TMD have been limited by narrow inclusion criteria, small sample sizes, and a lack of quantitative synthesis [3,17]. A 2018 review by Bueno et al. [3] concluded that evidence linking estrogen status to TMD was inconclusive, primarily due to methodological heterogeneity and small effect sizes. However, that review did not include recent high-quality studies such as Gaynor et al. (2021) [23] and Mursu et al. (2022) [24], which provide the most robust evidence to date. Our review extends previous work by incorporating recent studies, applying rigorous quality assessment with the Newcastle-Ottawa Scale, and evaluating the certainty of the evidence using GRADE criteria. Our findings suggest that, while evidence remains limited, there is now moderate-quality evidence supporting associations between hormonal contraceptive use, menopausal status, and TMD risk.

### 4.4. Biological Plausibility

The observed associations are biologically plausible given established mechanisms of estrogen action in the musculoskeletal and pain systems. Estrogen receptors are expressed in TMJ tissues, including synovium, fibrocartilage, and subchondral bone, where they regulate chondrocyte proliferation, collagen synthesis, and extracellular matrix turnover [5,6]. Estrogen also modulates inflammatory pathways by inhibiting nuclear factor-κB (NF-κB) signaling and reducing the production of pro-inflammatory cytokines such as IL-6 and TNF-α [37]. In the central nervous system, estrogen influences pain processing through interactions with serotonergic, GABAergic, and opioidergic systems and modulates descending pain-inhibition pathways [34]. Estrogen deficiency or dysregulation may therefore increase susceptibility to TMJ degeneration, inflammation, and central sensitization, all of which are implicated in TMD pathophysiology.

### 4.5. Clinical Implications

The findings of this review have several potential clinical implications. First, clinicians should be aware that hormonal factors, including hormonal contraceptive use and menopausal status, may influence TMD risk and symptom severity in female patients. Screening for hormonal exposures may aid in risk stratification and personalized treatment planning. Second, women experiencing TMD symptom exacerbation during specific phases of the menstrual cycle or following menopause may benefit from targeted interventions, including pain management strategies, physical therapy, and, potentially, hormonal therapies, although the latter requires further investigation. Third, the genetic evidence linking estrogen receptor polymorphisms to TMD susceptibility suggests that future research on precision medicine approaches that incorporate genetic profiling may improve risk prediction and therapeutic targeting.

However, it is important to emphasize that the current evidence does not support routine use of hormone replacement therapy or modification of hormonal contraceptive use solely for TMD management, as the benefits and risks of hormonal interventions must be carefully weighed in the context of each patient’s overall health profile.

The findings of this review may also have implications for headache-related rehabilitation strategies, particularly in conditions characterized by female predominance and hormonal modulation, such as migraine and cervicogenic headache. Shared trigeminal, cervical, and neuroinflammatory mechanisms may partly explain the frequent clinical overlap between TMD and headache disorders. These observations support the importance of integrated cervico-cranio-mandibular assessment and rehabilitation approaches in women presenting with overlapping pain conditions.

### 4.6. Strengths and Limitations

#### 4.6.1. Strengths

This systematic review has several strengths. First, we conducted a comprehensive literature search across multiple databases, including PubMed, Embase, Scopus, Web of Science, and Google Scholar, minimizing the risk of missing relevant studies. Second, we applied rigorous inclusion criteria that required validated TMD diagnostic criteria (RDC/TMD, DC/TMD, or equivalent), thereby enhancing the reliability and comparability of the findings. Third, we used the Newcastle-Ottawa Scale for quality assessment and GRADE criteria for certainty of evidence, providing transparent and standardized evaluation of study quality and evidence strength. Fourth, we included diverse hormonal exposures, encompassing hormonal contraceptive use, menopausal status, menstrual cycle variation, pregnancy, and genetic polymorphisms, offering a comprehensive overview of the role of estrogen in TMD.

#### 4.6.2. Limitations

Several limitations must be acknowledged. First, the small number of included studies (*n* = 7) and the limited number of high-quality studies constrain the strength of conclusions. Second, substantial heterogeneity in exposure definitions (e.g., self-reported vs. biochemically validated hormonal status), outcome measures (e.g., first-onset TMD vs. palpation pain vs. degenerative joint disease), and study designs (prospective cohort, cross-sectional, case–control) precluded quantitative meta-analysis. Third, most studies relied on self-reported hormonal exposures, which are subject to recall bias and misclassification. Fourth, few studies adjusted for important confounders such as body mass index, smoking, psychological factors (anxiety, depression), and pain catastrophizing, all of which are known to influence TMD risk and symptom severity. Fifth, the cross-sectional design of several included studies limits causal inference. Sixth, publication bias cannot be ruled out, as studies with null findings may be less likely to be published. Finally, all included studies were conducted in high-income countries (the United States, Finland, Iran, Italy, and Indonesia), limiting generalizability to other populations. Additionally, no region-specific databases (e.g., Chinese or Japanese databases) were searched, which may have limited the retrieval of potentially relevant non-English studies.

### 4.7. Implications for Future Research

Future research should prioritize well-designed prospective cohort studies with large sample sizes, biochemically validated hormonal assessments (including circulating estradiol, progesterone, and FSH levels), and standardized TMD diagnostic criteria (DC/TMD). Longitudinal studies tracking hormonal changes and TMD symptom trajectories across reproductive transitions (e.g., menarche, pregnancy, perimenopause, menopause) are needed to elucidate temporal relationships and causality. Interventional studies evaluating the effects of hormone replacement therapy, hormonal contraceptive formulations, and selective estrogen receptor modulators (SERMs) on TMD symptoms are warranted, with careful consideration of potential risks and benefits. Mechanistic studies investigating the molecular pathways linking estrogen signaling to TMJ inflammation, cartilage degradation, and pain processing will enhance understanding of pathophysiology and inform therapeutic targeting. Finally, incorporation of genetic profiling (e.g., estrogen receptor polymorphisms) and metabolomic analyses may facilitate precision medicine approaches and identify subgroups of women most likely to benefit from hormonal interventions.

## 5. Conclusions

This systematic review of seven studies provides moderate-quality evidence that hormonal factors, particularly hormonal contraceptive use and menopausal/climacteric status, are associated with increased risk and altered clinical features of temporomandibular disorders in women. High-quality studies demonstrated significant associations between hormonal contraceptive use and first-onset TMD, as well as between climacteric status and TMJ palpation pain, crepitus, and degenerative joint disease. Additional evidence supported variation in menstrual cycle-related symptoms and genetic susceptibility mediated by estrogen receptor polymorphisms. However, the limited number of studies, heterogeneity in exposure and outcome definitions, and reliance on self-reported exposures constrain definitive conclusions regarding causality and clinical implications.

Clinicians should consider hormonal factors when evaluating and managing female patients with TMD. Future research should prioritize large-scale prospective cohort studies that incorporate biochemically validated hormonal assessments, including real-time serum measurements of estradiol, progesterone, and follicle-stimulating hormone, correlated with clinical symptom trajectories and pain severity. Standardized diagnostic criteria, longitudinal monitoring across reproductive transitions, and comprehensive adjustment for relevant confounders are essential for clarifying causal relationships and improving evidence-based clinical decision-making. In addition, mechanistic and interventional studies investigating the effects of hormonal modulation on TMD symptoms may further support the development of precision medicine approaches for this complex and multifactorial disorder.

## Figures and Tables

**Table 1:** Characteristics of included studies.

First Author, Year	Study Design	Sample Size	Participant Age	TMD Diagnostic Criteria	Hormonal Exposure	Main Findings	Effect Estimate (95% CI)
Gaynor, 2021 [23]	Prospective cohort (OPPERA)	1475 women	Women aged 18–44 years	RDC/TMD with both self-reported symptoms and examiner-classified outcomes	Hormonal contraceptive use	HC use associated with increased self-reported TMD symptoms and first-onset pain symptoms; no significant association with examiner-classified TMD incidence	OR 1.37; OR 1.20; HR 1.36 (0.82–2.24)
Mursu, 2022 [24]	Cross-sectional (NFBC1966)	727 women (71 climacteric, 656 preclimacteric)	Mean age: 46 years	Modified DC/TMD	Climacteric status (amenorrhea > 4 months + FSH > 25 IU/L)	Associated with increased palpation pain, crepitus, and DJD	OR 2.64; OR 2.92; OR 2.27
LeResche, 2003 [25]	Prospective diary study	126 participants	Mean age: 29.4 years	Clinical TMD assessment	Menstrual cycle phase; oral contraceptive use	Cyclic variation in TMD symptoms	Not reported
Minervini, 2024 [26]	Case–control study	67 women	Women aged 18–40 years	DC/TMD	Pregnancy status	Lower chronic pain grades during pregnancy	Not reported
Ribeiro-Dasilva, 2009 [27]	Case–control genetic association	300 women	Women aged 18–60 years	RDC/TMD	ERα gene polymorphisms	Associated with painful and painless TMJD	OR 3.20; OR 2.51
Rosanto, 2020 [28]	Case–control study	40 postmenopausal women	Postmenopausal women	RDC/TMD	Serum estradiol levels	Higher mean estradiol in the ADD group, not significant	*p* > 0.05

Footnote: Six clinical studies are presented in Table 1. One additional mechanistic study [29] was included as supportive biological evidence and is described in Section 4 but was not considered part of the main clinical synthesis. TMD, temporomandibular disorders; TMJ, temporomandibular joint; RDC/TMD, Research Diagnostic Criteria for TMD; DC/TMD, Diagnostic Criteria for TMD; HC, hormonal contraceptive; OR, odds ratio; CI, confidence interval; NOS, Newcastle-Ottawa Scale; OPPERA, Orofacial Pain: Prospective Evaluation and Risk Assessment; NFBC1966, Northern Finland Birth Cohort 1966; FSH, follicle-stimulating hormone; DJD, degenerative joint disease; IL-6, interleukin-6.

**Table 2:** Risk of Bias Assessment (Newcastle-Ottawa Scale).

First Author, Year	Selection (0–4 Stars)	Comparability (0–2 Stars)	Outcome/Exposure (0–3 Stars)	Total Score	Quality Rating
Gaynor, 2021 [23]	4	2	2	8/9	High
Mursu, 2022 [24]	4	2	2	8/9	High
LeResche, 2003 [25]	4	2	1	7/9	High
Minervini, 2024 [26]	3	1	2	6/9	Moderate
Ribeiro-Dasilva, 2009 [27]	4	2	1	7/9	High
Rosanto, 2020 [28]	2	1	2	5/9	Moderate

**Table 3:** Summary of evidence by hormonal exposure category.

Hormonal Exposure	Number of Studies	Quality of Evidence	Main Findings	GRADE Certainty
Hormonal contraceptive use	2	2 high-quality clinical studies	Increased risk of self-reported first-onset TMD pain symptoms (OR 1.37, 95% CI 1.13–1.66) and concurrent self-reported TMD symptoms (OR 1.20, 95% CI 1.06–1.35); no significant association with examiner-classified incident TMD (HR 1.36, 95% CI 0.82–2.24); cyclic symptom variation independent of oral contraceptive use	Moderate
Menopausal/climacteric status	2 clinical studies	1 high, 1 moderate	Increased risk of palpation pain, crepitus, and degenerative joint disease; non-significantly higher estradiol levels in anterior disc displacement	Moderate
Menstrual cycle variation	1	1 high-quality clinical study	Cyclic variation in TMD symptoms, with peak severity during menstruation and secondary peak at ovulation	Low
Pregnancy	1	1 moderate-quality clinical study	Lower chronic pain grades in pregnant women; significance lost after multiple-comparison correction	Very low
Estrogen receptor gene polymorphisms/mechanistic evidence	2 (1 clinical + 1 mechanistic supportive)	1 high-quality clinical + 1 supportive mechanistic study	ERα polymorphisms associated with painful and painless TMJD; mechanistic evidence supports estrogen-mediated modulation of IL-6 inflammatory pathways relevant to TMD	Low

TMD, temporomandibular disorders; TMJD, temporomandibular joint disorder; OR, odds ratio; CI, confidence interval; NOS, Newcastle-Ottawa Scale; GRADE, Grading of Recommendations Assessment, Development and Evaluation; OC, oral contraceptive; ERα, estrogen receptor-α; IL-6, interleukin-6.

## Data Availability

All data analyzed in this review are included in the article. No new data were created or analyzed in this study.

## Supplementary Materials

The following supporting information can be downloaded at:  https://www.mdpi.com/article/10.3390/ijerph23060717/s1, Table S1: PRISMA 2020 Checklist [20]; Table S2: Full Search Strategies for All Databases.
