# Supplementary material for: Estrogen Status and Temporomandibular Disorders: A Systematic Review
Source: Int J Environ Res Public Health. 2026 May 28;23(6):717. doi: 10.3390/ijerph23060717 (PMC13300506; doi:10.3390/ijerph23060717)
Supplement: Supplementary file 1 [file ijerph-23-00717-s001.zip › ijerph-4264756-Table S2 Full Search Strategies for All Databases.pdf]

Supplementary Table S2. Full Search Strategies for All Databases

Database	Search Strategy
**PubMed/MEDLINE**	(“temporomandibular joint disorders”[MeSH] OR “temporomandibular disorder”[tiab] OR ”TMD”[tiab] OR ”temporomandibular joint dysfunction”[tiab] OR ”TMJ disorder”[tiab] OR “craniomandibular disorder”[tiab]) AND (”estrogens”[MeSH] OR ”estrogen”[tiab] OR ”estradiol”[tiab] OR ”hormone replacement therapy”[MeSH] OR ”HRT”[tiab] OR ”menopause”[MeSH] OR ”menopause”[tiab] OR ”postmenopause”[tiab] OR ”climacteric”[tiab] OR ”contraceptives, oral”[MeSH] OR ”oral contraceptive”[tiab] OR “hormonal contraceptive*“[tiab] OR”menstrual cycle”[MeSH] OR “menstrual cycle”[tiab] OR “pregnancy”[MeSH] OR “pregnancy”[tiab] OR “estrogen receptor”[tiab] OR “ERα”[tiab])
**Embase**	(‘temporomandibular joint disorder’/exp OR ‘temporomandibular disorder’:ti,ab OR ’TMD’:ti,ab OR ’temporomandibular joint dysfunction’:ti,ab OR ’TMJ disorder’:ti,ab OR ‘craniomandibular disorder’:ti,ab) AND (’estrogen’/exp OR ’estradiol’/exp OR ’estrogen’:ti,ab OR ’estradiol’:ti,ab OR ’hormone replacement therapy’/exp OR ’HRT’:ti,ab OR ’menopause’/exp OR ’menopause’:ti,ab OR ’postmenopause’:ti,ab OR ’climacteric’:ti,ab OR ’oral contraceptive’/exp OR ’oral contraceptive’:ti,ab OR ’hormonal contraceptive*‘:ti,ab OR ’menstrual

Database	Search Strategy
	cycle'/exp OR 'menstrual cycle':ti,ab OR 'pregnancy'/exp OR 'pregnancy':ti,ab OR 'estrogen receptor':ti,ab OR 'ERα':ti,ab)
**Scopus**	TITLE-ABS-KEY("temporomandibular disorder*" OR "TMD" OR "temporomandibular joint dysfunction" OR "TMJ disorder*" OR "craniomandibular disorder") AND TITLE-ABS-KEY (estrogen OR estradiol OR "hormone replacement therapy" OR HRT OR menopause OR postmenopause OR climacteric OR "oral contraceptive" OR "hormonal contraceptive*" OR "menstrual cycle" OR pregnancy OR "estrogen receptor" OR "ERα")*
**Web of Science**	TS=("temporomandibular disorder*" OR "TMD" OR "temporomandibular joint dysfunction" OR "TMJ disorder*" OR "craniomandibular disorder*") AND TS=(estrogen OR estradiol OR "hormone replacement therapy" OR HRT OR menopause OR postmenopause OR climacteric OR "oral contraceptive*" OR "hormonal contraceptive*" OR "menstrual cycle" OR pregnancy OR "estrogen receptor" OR "ERα")
**Google Scholar**	"temporomandibular disorders" estrogen OR estradiol OR menopause OR hormonal contraceptives OR menstrual cycle OR pregnancy OR estrogen receptor

Abbreviations: TMD, temporomandibular disorders; TMJ, temporomandibular joint; HRT, hormone replacement therapy; ERα, estrogen receptor alpha.

Search Period: Database searches were conducted from database inception through September 2025.
